# Morphological embryo selection: an elective single embryo transfer proposal

**DOI:** 10.5935/1518-0557.20180015

**Published:** 2018

**Authors:** Francisco Parera Déniz, Carlos Encinas, Jorge La Fuente

**Affiliations:** 1 Embriovid, La Paz, Bolivia

**Keywords:** elective single embryo transfer, morphological embryo selection, implantation rate, multiple pregnancy rate.

## Abstract

**Objective:**

To describe a patient selection method for elective single embryo transfer (eSET), emphasizing inclusion criteria and results.

**Methods:**

This retrospective study included all cases seen in a private clinic between June 2011 and December 2016, in La Paz, Bolivia (3600 meters above sea level). Elective single embryo transfer was the method of choice in 34 IVF/ICSI cycles, all in the blastocyst stage. Gardner's blastocyst classification criteria were used. Between the two stages of the study (July 2015), each embryo grade implantation rate was recalculated, which led to the expansion of the inclusion criteria.

**Results:**

The clinical pregnancy rate of the 34 cases in the first transfer group was 55.9% (19/34). Twin or multiple pregnancies did not occur. The cumulative pregnancy rate to date is 64% [(19+3)/34]. The first stage comprised 2.56% (12/468) of the patients offered elective single embryo transfers; the implantation rate was 58.3% (7/12). In the second stage, 14.29% (22/154) of the patients were eligible, and the implantation rate was 54.55% (12/22).

**Conclusion:**

The implementation of an eSET program based on in-depth morphological embryo assessment combined with the calculation of the implantation potential of each embryo grade led to acceptable clinical outcomes and fewer multiple pregnancies in patients transferred two embryos. Each clinic should be aware of the implantation rates of each embryo grade in its own setting.

## INTRODUCTION

Twin and multiple pregnancies are the direct consequence of the number of transferred embryos, and yield higher risk of intrauterine growth restriction, premature birth, and perinatal morbidity and mortality (Pinborg, 2005). Since 1996, transfers of more than three embryos have declined considerably in Latin America. However, they still account for 2.5% of all cycles. Transfers of three embryos have not decreased in number and still account for approximately 20% of all transfers (Zegers-Hochschild *et al*., 2011). Consequently, a significant increase in the proportion of transfers of two embryos has been observed. The number of multiple pregnancies of higher grade, i.e., with three or more gestational sacs, has decreased, although twin pregnancies still occur in more than 20% of the cases (Zegers-Hochschild *et al*., 2016).

Decreasing the number of three or more transferred embryos to two is a relatively simple decision, since it has been clearly shown that transferring more than two embryos does not increase pregnancy rates (PR) (Templeton & Morris, 1998). Decreasing twin pregnancy rate requires the implementation of an elective single embryo transfer (eSET) program. If performed with embryo morphological assessment, it may affect the success rate of the procedure (Gerris & De Sutter, 2009).

Nowadays, the transfer of one elective embryo comprises the use of two available technologies, blastocyst culture and vitrification. After successful blastocyst culture, the transfer method is selected. Morphological blastocyst assessment has been challenged by technologies such as preimplantation genetic screening (PGS). Trophectoderm biopsies and molecular techniques such as next-generation sequencing (NGS) have granted PGS promising results (Sermon *et al*., 2016). Nevertheless, access to high-complexity assisted reproduction technologies in Latin America is problematic, mainly in what concerns the cost of the procedures. The vast majority of the cycles in Latin America are not covered by public healthcare systems or reimbursed by private health insurance.

**Figure 1:**
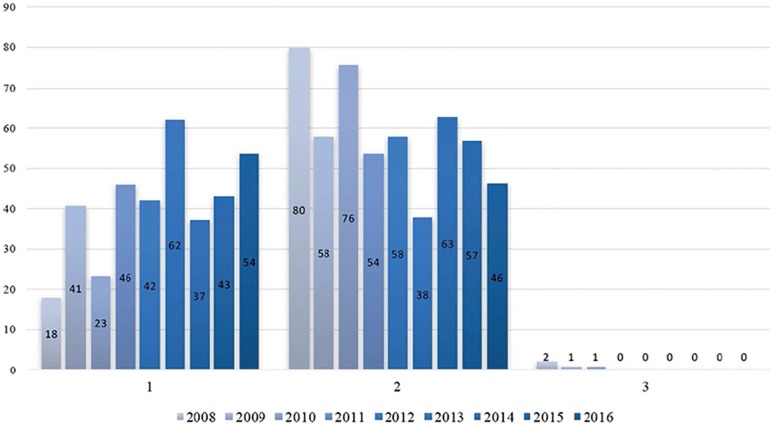
Number of transferred embryos (%).

**Figure 2:**
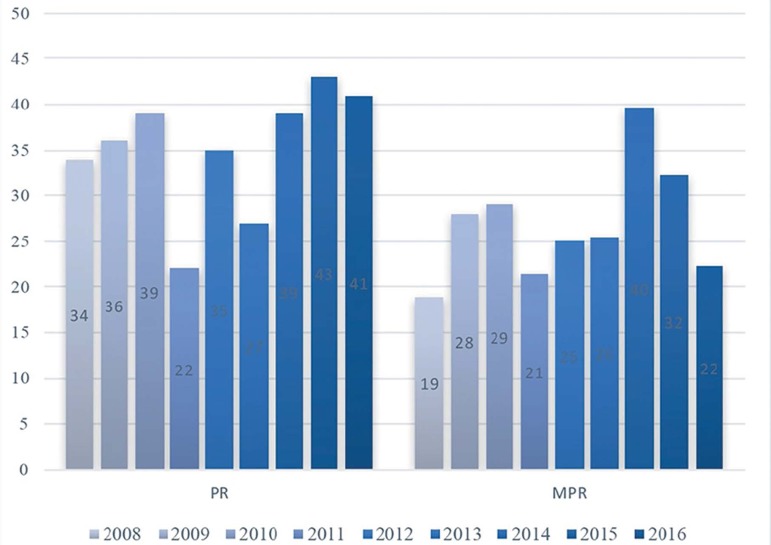
Pregnancy and Multiple Pregnancy rates (twins or higher) (%).

Data from the Latin American Registry of Assisted Reproduction reveal that PGS and blastocyst stage transfers are offered to fewer than 20% of the patients, while cleavage stage transfers still account for the majority of the cases reported (Zegers-Hochschild *et al*., 2016). The above and our impressions indicate that morphological selection will continue to be a mainstay in blastocyst selection in Latin America over the next years. Since 2008, the proportion of twin pregnancies in our clinic has been greater than 20% (40% in 2014), although most transfers involved one or two embryos (99.7%) (Figures 1 and 2). Consequently, in 2011 we embarked on an eSET program based exclusively on the morphological characteristics of the embryos available at the time of transfer (day +5 or +6).

The aim of this work was to demonstrate the selection criteria used in the program and some of its results.

## MATERIAL AND METHODS

Ovulation induction was performed with GnRH agonist (Lupron^®^) long protocol and uFSH/LH (Menopur^®^) step-down regimen for 10 days, followed by transvaginal follicular aspiration on day 13. Endometrial preparation was carried out with Decapeptyl depot^®^, 15 days before the administration of micronized estradiol for 18 days. After that, luteal phase support was started with Micronized Progesterone 400 mg (Progendo^®^) administered vaginally twice a day or progesterone rings (Fertiring^®^)

IVF and ICSI cycles were performed as described in the literature. Insemination or injection procedures were carried out five hours after oocyte pick-up. In all cycles the embryos were cultured according to the protocols recommended by culture media suppliers (Life Global). Given that the laboratory is located approximately 3600 meters above sea level, the CO_2_ proportion was adjusted to attain a pH of 7.3. Two types of incubators were used: conventional water-jacketed CO_2_ (Thermo Forma) incubators and tri-gas (CO_2_/N_2_) bench top incubators (K-Systems).

Embryo assessment was performed in IX71 and IX73 inverted microscopes, equipped with Nomarski DIC optics, Relief Contrast Optics, and digital imaging software (Olympus, Japan). At least two embryologists were present at the moment of blastocyst evaluation; the final grade given to an embryo was determined by consensus between them. Gardner's classification criteria were used (Gardner *et al*., 2000).

Patients meeting the inclusion criteria were informed of the advantages and disadvantages of joining the study, and the individuals who agreed to join the study signed informed consent terms. The only variable taken into account in the eSET program was the quality of the embryos at the time of transfer (D+5 or D+6). Donor (15/34) and autologous (19/34) oocytes were included.

In the first stage of the study (June 2011 to July 2015), the inclusion criteria were established according to the patients' three top-quality embryos, i.e., first and second embryos graded at least as 3AA, and third embryos graded at least as 2AA. In the second stage (August 2015 to December 2016), the inclusion criteria was expanded to include first embryos graded 3AA, second embryos graded at least as 2AB or 2BA, and third embryos graded at least as cavitating morulas.

The implantation rate of each of embryo grade was determined based on all cycles, in which 0 or 100% implantation was attained; in other words, all one-embryo and two-embryo transfers in which all or none implanted. A total of 1599 embryos were analyzed.

A chi-square test was performed on Systat 13 to verify the frequency distributions of the different embryo grades with known (implanted or not) versus unknown outcomes (two were transferred but only one implanted). Differences with *p* values <0.05 were considered significant. The same analysis was used to compare the embryos with better implantation rates versus the embryos transferred in the eSET program.

## RESULTS

The first stage comprised 2.56% (12/468) of the patients included in the eSET program; the implantation rate (IR) was 58.3% (7/12).

**Figure 3:**
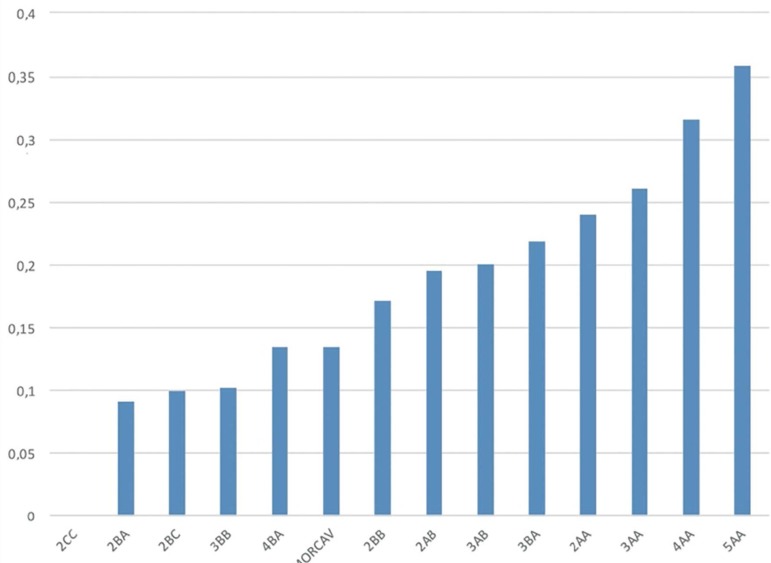
Implantation rates of multiple embryo morphological scores (only categories with a sample size n=10 were considered).

Table 1 shows the implantation rates calculated for each embryo grade. Figure 3 shows the grades with at least ten embryos.

**Table 1:** Implantation Rate of all embryo grades and eSET program embryos.

GRADE	IMPLANT	NO IMPLANT	TOTAL	IR %
**1AA**	0	1	1	0.0%
**1CA**	0	1	1	0.0%
**2CA**	0	1	1	0.0%
**4CC**	0	1	1	0.0%
**5AB**	0	1	1	0.0%
**1BB**	0	2	2	0.0%
**1CB**	0	2	2	0.0%
**3CB**	1	1	2	50.0%
**5BA**	0	2	2	0.0%
**4CB**	0	3	3	0.0%
**1CC**	1	3	4	25.0%
**3CA**	0	4	4	0.0%
**MOR**	0	4	4	0.0%
**3AC**	2	3	5	40.0%
**2CB**	0	6	6	0.0%
**2AC**	1	7	8	12.5%
**4AB**	1	7	8	12.5%
**4BB**	1	7	8	12.5%
**2CC**	0	10	10	0.0%
**2BA**	3	30	33	9.1%
**2BC**	1	9	10	10.0%
**3BB**	7	62	69	10.1%
**4BA**	2	13	15	13.3%
**MORCAV**	37	239	276	13.4%
**2BB**	14	68	82	17.1%
**2AB**	25	103	128	19.5%
**3AB**	17	68	85	20.0%
**3BA**	12	43	55	21.8%
**2AA**	47	149	196	24.0%
**3AA**	109	309	418	26.1%
**4AA**	35	76	111	31.5%
**5AA**	5	9	14	35.7%
**eSET**	19	15	34	55.9%

**Figure 4:**
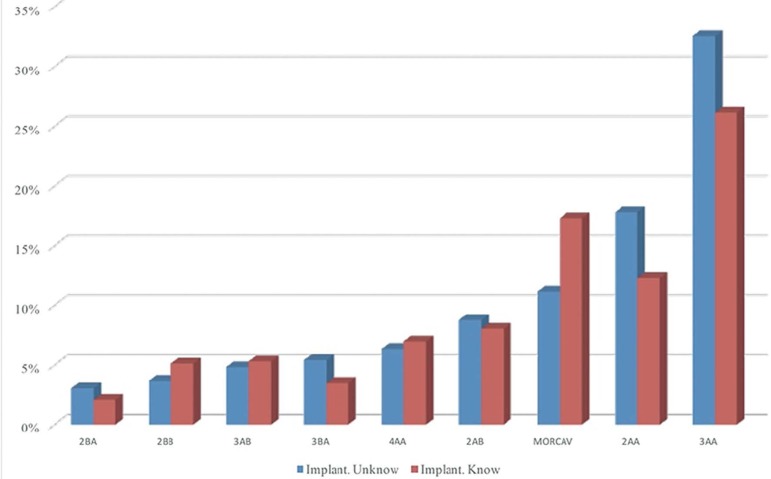
IR calculation. Included vs. not included. Distribution frequency when implantation of each embryo is known or unknown.

The comparison of the embryos included in the calculation of the implantation rate for each grade vs. embryos not included (outcome unknown for each embryo) did not reveal significant differences (chi-square 20,053 - *p*=0,01) (Figure 4).

In the second stage 14.29% (22/154) of the patients were eligible, and the implantation rate was 54.55% (12/22).

The overall pregnancy rate of the 34 cases in the first transfer was 55.9% (19/34). Twin and multiple pregnancies were not observed. The cumulative pregnancy rate to date is 64% [(19+3)/34].

**Figure 5:**
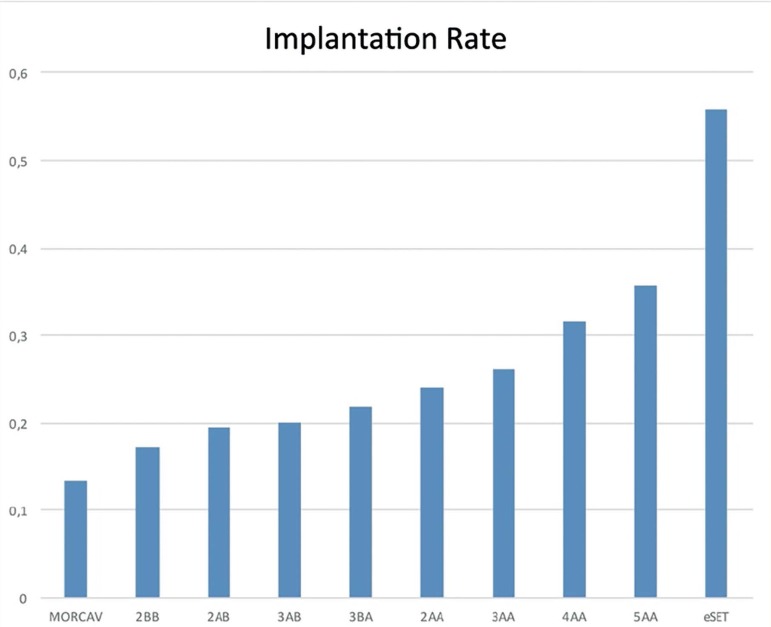
Implantation rate of top-quality and eSET embryos.

The chi-square test comparing the implantation rates for the top performing grades versus the rates observed in eSET embryos yielded the following *p*-values: vs. 5AA, 0.2; vs. 4AA, 0.01; vs. 3AA, 0.0002; vs. 2AA, 0.0001 (Figure 5).

The pregnancy rates of all cycles in which two embryos were transferred versus all cycles including eSET were not significantly different (36.5% vs. 39%, chi-square *p*=0.499).

## DISCUSSION

High twin pregnancy rates in two-embryo transfers imply an at least acceptable level of embryo quality, expressed in the realization of their implantation potential. Lowering twin pregnancy rates without compromising the overall pregnancy rate can only be achieved with an eSET program. Two fundamental features are required in successful eSET programs: good inclusion criteria and strict selection of the most competent embryos (Gerris & De Sutter, 2009; Ombelet, 2016). Technologies such as PGS allow for strict embryo selection and the differentiation of embryos that would otherwise seem similar through the eyes of other technologies.

Morphological selection of blastocyst stage embryos is apparently less strict, and therefore leads to poorer outcomes. However, since there are different grading systems, not all relate to outcomes with the same strength. A selection system should approach as closely as possible the calculated implantation rate for each embryo type or grade, with the achieved one replacing an embryo of this grade. Given the low impact of PGS in Latin America (1071/55840 blastocyst stage cycles in 2013 with PGD and PGS) (Zegers-Hochschild *et al*., 2016), presumably because of the cost of performing it, the possibilities of morphological embryo selection should be further analyzed.

The blastocyst classification system proposed by Gardner *et al*. (2000) has attained significant use in ART laboratories. However, it should be mentioned that not all laboratories categorize their embryos equally, as a result of the subjective nature of the system. In addition, not all extended culture conditions result in equal implantation rates for each embryo grade. Therefore, it is essential that each laboratory objectively assess its own embryo implantation rates.

One of the peculiarities of our patient selection method is its particularly embryocentric perspective, based exclusively on blastocyst morphology. Female patient age is not an eligibility criterion, since it is reflected in embryo quality. If the inclusion criteria are too strict (first stage), very few cases might end up being included in the program. On the other hand, if the criteria are too flexible, success rates may drop. A discussion that remains open is whether, in order to determine the cut-off level of the inclusion criteria, pregnancy rates or twin pregnancies should be prioritized. 

In our case, decreasing the selection pressure in the second stage seems to have reduced twin pregnancies from 40% to 22% without a significant decrease in pregnancy rates (58.3% to 54.6%). Our goal was to bring twin pregnancies closer to 10%. A pregnancy rate of 55.9% (when replacing one embryo pregnancy rate is equal to implantation rate) in the first transfer, without twin pregnancies, satisfied our expectations. The eSET program did not seem to decrease the overall clinical pregnancy rate itself. The outcomes of all two-embryo transfers including and excluding eSET cycles were compared and no differences were found.

Interestingly, the blastocysts replaced in the eSET cycles had a higher implantation rate than some embryos with higher implantation potential (4AA - 3AA - 2AA) (33/34 transfers). Two possible explanations were considered. The first was that this finding may have occurred due to the fact that the two groups were not comparable, since in eSET all transferred embryos were included, while when each embryo grade implantation potential was calculated the embryos with unknown implantation statuses (two embryos transferred but only one implanted) were not included. The second was that the inclusion criteria took into account the top three embryos with high implantation potential, possibly introducing greater selective pressure.

These questions could have been answered if all embryos had been fingerprinted, a measure not within our possibilities at this time.
